# Cortical type: a conceptual tool for meaningful biological interpretation of high-throughput gene expression data in the human cerebral cortex

**DOI:** 10.3389/fnana.2023.1187280

**Published:** 2023-06-22

**Authors:** Ariadna Sancha-Velasco, Alicia Uceda-Heras, Miguel Ángel García-Cabezas

**Affiliations:** ^1^Department of Anatomy, Histology and Neuroscience, School of Medicine, Autonomous University of Madrid, Madrid, Spain; ^2^Master Program in Neuroscience, Autonomous University of Madrid, Madrid, Spain; ^3^Ph.D. Program in Neuroscience UAM-Cajal, Autonomous University of Madrid, Madrid, Spain; ^4^Neural Systems Laboratory, Department of Health Sciences, Boston University, Boston, MA, United States

**Keywords:** human neuroanatomy, biological significance, cerebral cortex, excitation, inhibition, epigenetic regulation, mesocortex, eulaminate

## Abstract

The interpretation of massive high-throughput gene expression data requires computational and biological analyses to identify statistically and biologically significant differences, respectively. There are abundant sources that describe computational tools for statistical analysis of massive gene expression data but few address data analysis for biological significance. In the present article we exemplify the importance of selecting the proper biological context in the human brain for gene expression data analysis and interpretation. For this purpose, we use cortical type as conceptual tool to make predictions about gene expression in areas of the human temporal cortex. We predict that the expression of genes related to glutamatergic transmission would be higher in areas of simpler cortical type, the expression of genes related to GABAergic transmission would be higher in areas of more complex cortical type, and the expression of genes related to epigenetic regulation would be higher in areas of simpler cortical type. Then, we test these predictions with gene expression data from several regions of the human temporal cortex obtained from the Allen Human Brain Atlas. We find that the expression of several genes shows statistically significant differences in agreement with the predicted gradual expression along the laminar complexity gradient of the human cortex, suggesting that simpler cortical types may have greater glutamatergic excitability and epigenetic turnover compared to more complex types; on the other hand, complex cortical types seem to have greater GABAergic inhibitory control compared to simpler types. Our results show that cortical type is a good predictor of synaptic plasticity, epigenetic turnover, and selective vulnerability in human cortical areas. Thus, cortical type can provide a meaningful context for interpreting high-throughput gene expression data in the human cerebral cortex.

## 1. Introduction

In the last decades, transcriptomic studies using high-throughput gene expression techniques, like microarrays and next-generation sequencing, have allowed the study of gene expression activity of thousands of genes simultaneously (Reddy and Ramanujam, 2018; Carangelo et al., 2022). Older techniques, like Sanger sequencing, *in situ* hybridization, and immunohistochemistry, could quantify or detect the expression of one or some few genes in biological tissues (Liu et al., 2013; Mills et al., 2017), making the characterization of cellular and tissular genetic profiles a slow and resource consuming process. In contrast, the contemporary high-throughput gene expression techniques yield massive data that have revolutionized molecular biology and are also impacting human neuroscience (Leung et al., 2021; Chen et al., 2022; Chiou et al., 2022). These techniques are providing new cellular ontologies in the primate brain (*e. g.*, He et al., 2021) and also help unveiling the molecular characterization of human diseases (*e. g.*, Gu et al., 2022).

The interpretation of the massive data obtained using high-throughput gene expression techniques is challenging and requires two types of analysis. First, the computational analysis to identify statistically significant differences; and second, the “biological” analysis to identify biologically significant differences. The computational analysis is challenging because of the large amount of data that must be handle by researchers, who require adequate training and specialization (Bhandari et al., 2022); fortunately, researchers count with abundant manuals and reviews that describe computational and informatic tools used for statistical analyses of massive gene expression data (*e. g.*, Kappelmann-Fenzl, 2021). In contrast, the articles that address how to analyze data for biological significance (*e. g.*, Olson, 2006; Davidson, 2015) are scarce, even though statistical significance should be computed considering biologically meaningful contexts.

Ideally, high-throughput gene expression techniques should be applied to tissue samples following a careful experimental design that will affect the downstream analysis of the data. The goal in most gene expression studies in the human brain should be identifying genes that are differentially expressed between two or more groups of individuals (control vs. disease), and/or brain regions (cingulate cortex vs. nucleus accumbens), and/or life cycle stages (embryo vs. adult). In any case, the adequate selection of brain regions of interest (ROIs) before processing is a key step that, in the end, will facilitate or obscure drawing biologically meaningful conclusions. For instance, if we are looking for gene expression differences in the cerebral cortex of autistic vs. control subjects it would be of interest to compare samples from limbic mesocortical areas, like the anterior cingulate cortex, with samples from highly eulaminate areas, like the primary visual area, because the former are selectively affected in autism spectrum disorders and the latter are preserved (Zikopoulos and Barbas, 2010; Zikopoulos et al., 2018). In spite of this, most studies of gene expression using high-throughput techniques in the brain (and other tissues) are performed under “pure observation” experimental designs that are not based on hypothesis or biological context, an experimental approach called “Discovery Science” (Davidson, 2015). Alternatively, a meaningful biological context will help designing experiments based on testable predictions and aiming at unveiling causal explanations for the observed regularities in the data. In neurobiology, causal explanations are obtained (or at least, approached) when data is examined from the points of view of development and evolution; these points of view allow to identify the molecular mechanisms that give rise to structure and synaptic circuits in adult brains (Davidson, 2015; Nieuwenhuys and Puelles, 2016; for a practical example see Puelles, 2022). Thus, in principle, a meaningful biological context for the interpretation of massive gene expression data in the human brain should provide testable predictions based on the ontogeny and phylogeny of the brain structures that are sampled for high-throughput gene expression techniques.

Examples of contexts for meaningful biological interpretation of human cerebral cortex data that provide testable predictions are the Tetrapartite Model of Pallial Development and Evolution (Puelles et al., 2019), the Updated Hypothesis on the Dual Origin of the Neocortex (García-Cabezas et al., 2022), and the Structural Model for cortico-cortical connections (Barbas, 1986; Barbas and Rempel-Clower, 1997; García-Cabezas et al., 2019). The Tetrapartite Model of Pallial Development and Evolution and the Updated Hypothesis on the Dual Origin of the Neocortex provide (hypothetical) causal mechanisms for the tangential expansion during evolution of the human (and mammalian) cerebral cortex that are compatible with the specification of pallial sectors during embryonic development; on the other hand, the Structural Model predicts the laminar pattern of cortico-cortical connections between two given cortical areas (and thus, their relative position along cortical hierarchies) in non-human primates (and other mammals). The predictions advanced from these theoretical frameworks (Barbas and Rempel-Clower, 1997; García-Cabezas et al., 2019, 2022; Puelles et al., 2019; Aparicio-Rodríguez and García-Cabezas, 2023) are based on the gradual and systematic complexification of laminar architecture along the human (and mammalian) cerebral cortex. Such gradual complexification of cortical laminar architecture is conceptualized as Cortical type, defined as “the constant variations that one observes in each of the layers in different regions” of the cortex (von Economo and Koskinas, 1925/2008; von Economo, 1927/2009). Cortical types can be defined and mapped on the human cerebral cortex allowing for extrapolation to humans of predictions based on the Tetrapartite Model of Pallial Development, the Updated Hypothesis on the Dual Origin of the Neocortex, and the Structural Model (García-Cabezas et al., 2020, 2022).

The aim of the present article is to exemplify the importance of selecting the proper biological context in the human brain for gene expression data analysis and interpretation. For this purpose, we made testable predictions about gene expression in areas of the human temporal cortex based on cortical type variation (García-Cabezas et al., 2020). Cortical type has been used before to guide hypothesis driven experiments in non-human primates (Box 1). Our hypotheses were that the expression of several genes would vary gradually along the gradient of cortical types. First, the expression of genes related to glutamatergic transmission would be higher in areas of simpler cortical type because the neuropil in these areas is probably enriched in excitatory synapses compared to the eulaminate neuropil (see Box 1). Second, the expression of genes related to GABAergic transmission would be higher in areas of more complex cortical type because these areas have more inhibitory Parvalbumin expressing interneurons than mesocortical areas (see Box 1). Third, the expression of genes related to epigenetic regulation, like enzymes that modify histones, would be higher in areas of simpler cortical type because these areas are likely endowed with higher synaptic plasticity (see Box 1) and, as suggested by Barbas (1995), require more flexibility to perform their functions related to learning and memory. Thus, this third hypothesis is based on relating flexible neuron function and higher synaptic plasticity to flexible regulation of gene expression in the nucleus of neurons (and, probably, of glial cells).

**BOX 1 Example of hypothesis-driven studies based on cortical type.**

Cortical type has been used as a theoretical tool to advance hypotheses on the structure and function of cortical areas in non-human primates. For instance, Helen Barbas proposed that mesocortical areas “retain some features observed in ontogeny, which may explain their great plasticity and involvement in learning and memory, but also their preferential vulnerability in several psychiatric and neurologic disorders” (Barbas, 1995). Using this conceptual framework, Barbas and her associates gathered *postmortem* anatomical data from macaque brains showing that the expression of markers that inhibit synaptic plasticity (like myelin, perineuronal nets, and Parvalbumin expressing interneurons) is higher in eulaminate (more complex laminar architecture) than in mesocortical (simpler laminar architecture) areas (Dombrowski et al., 2001; Barbas and García-Cabezas, 2015; García-Cabezas et al., 2017; Joyce et al., 2020; John et al., 2022), while the expression of markers that favor synaptic plasticity (like the enzyme calcium/calmodulin-dependent protein kinase II; CaMKII) is higher in mesocortical than in eulaminate areas (García-Cabezas et al., 2017). ***These anatomical findings suggest that synaptic plasticity in the primate cerebral cortex may decrease in parallel to laminar complexity***. Barbas and her associates also showed that mesocortical areas in macaques have lower neuron density than eulaminate areas (Dombrowski et al., 2001); lower neuron density together with smaller amount of intracortical myelin in mesocortical than in eulaminate areas (Barbas and García-Cabezas, 2015; García-Cabezas et al., 2017; John et al., 2022) could imply that there is more room for synapses in the mesocortical than in the eulaminate neuropil, which is compatible with data obtained in macaques showing longer basolateral dendrites and more spines in mesocortical than in eulaminate pyramidal neurons [Elston et al., 2005; see also Table 2 in García-Cabezas et al. (2018)]. Most of those synapses may be excitatory because they are the dominant type of synapse in the cerebral cortex (Peters et al., 1991). ***These anatomical findings suggest that the number of excitatory synapses in the primate cortical neuropil may decrease in parallel to laminar complexity***.

To test these hypotheses, we chose the human temporal cortex because it has a variety of areas that differ in function (Mesulam, 2023) and vulnerability to Alzheimer’s disease (Braak and Braak, 1996), probably in relation to differences in cortical type. Then, we data-mined an open-source data base (Allen Human Brain Atlas; Hawrylycz et al., 2012; Shen et al., 2012) to collect gene expression data from several regions of the human temporal cortex and analyzed them to confirm or reject our predictions. We also collected gene expression data from the caudate nucleus for comparison with the temporal cortex because the relative homogeneity of caudate neurons and synaptic connections [reviewed in Del Rey and García-Cabezas (2023)] contrasts with the variety of the neuron types, layers, and circuits of the temporal cortex (Sanides, 1975; Hoistad and Barbas, 2008; Benavides-Piccione et al., 2021). We found that the expression of most analyzed genes did not show statistically significant differences across temporal areas, but some did show differences in agreement with the predicted gradual expression along the laminar complexity gradient of the human cortex. Our results show that cortical type can provide a meaningful context for interpreting high-throughput gene expression data in the human cerebral cortex.

## 2. Materials and methods

### 2.1. Experimental design

**FIGURE 1:**
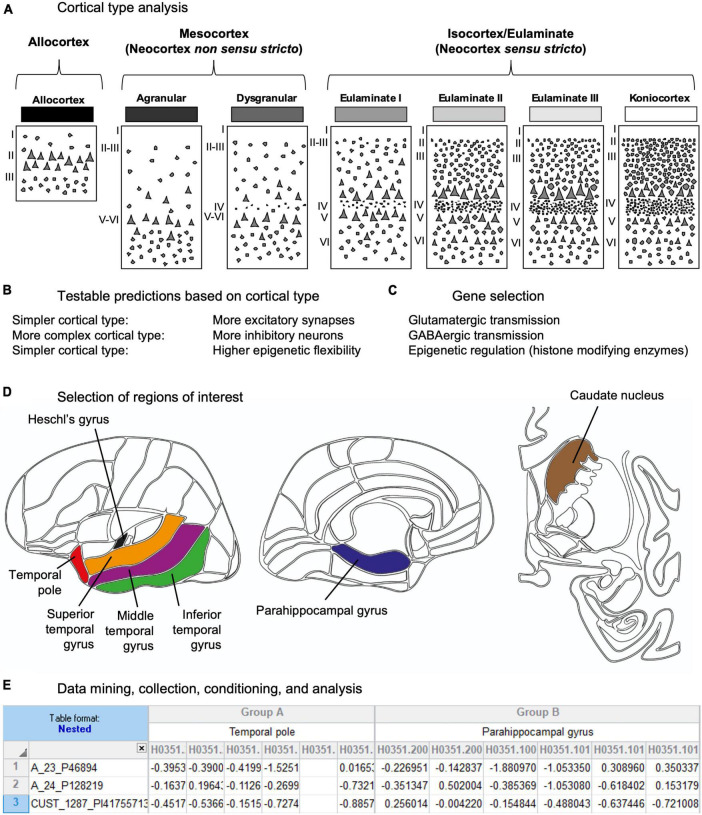
Experimental design. **(A)** The first step in this study was cortical type analysis of the human temporal cortex. Cartoons depict human cortical types according to García-Cabezas et al. (2020). Laminar architecture complexity increases progressively from allocortical (left) to koniocortical (right) areas. All cortical types (allocortical, mesocortical agranular, mesocortical dysgranular, eulaminate I, eulaminate II, eulaminate III, and koniocortex) are repesented in greyscale (black: simplest laminar architecture; white: most complex laminar architecture). **(B)** The second step in this study was making testable predictions of gene expression across temporal areas based on cortical type analysis. **(C)** The third step in this study was selecting genes to test the predictions. **(D)** The fourth step in this study was selecting the ROIs in the Allen Human Brain Atlas. The left cartoon shows the selected cortical ROIs in a lateral view of the left-brain hemisphere; the center cartoon shows the selected cortical ROIs in a medial view of the left-brain hemisphere; the right cartoon shows the caudate ROI in a coronal section of the brain. **(E)** Finally, the last steps of this study were data mining, collection, conditioning, and analysis using Excel spreadsheets.

In the present article we analyzed gene expression data in the human temporal cortex from the Allen Brain Institute Human Brain Atlas Database that were obtained using microarrays in postmortem human brain samples (Hawrylycz et al., 2012; Shen et al., 2012). First, we chose cortical type as the conceptual tool for advancing testable hypotheses on gene expression data in the human cerebral cortex (Figure 1A). Contrary to cortical areas, which are arbitrary parcellations of the cortex based on cytoarchitectonic features (Amunts and Zilles, 2001), cortical types are embedded within the mammalian body developmental plan (García-Cabezas et al., 2022) and are related to synaptic plasticity (García-Cabezas et al., 2017) and the laminar patterns of cortico-cortical connections (García-Cabezas et al., 2019). Second, based on cortical type analysis of human temporal areas and extrapolation of non-human primate data, we advanced several testable predictions on gene expression (Figure 1B). Third, we selected genes to test the predictions; these genes were related to glutamatergic transmission, GABAergic transmission, and epigenetic regulation (histone-modifying enzymes) (Figure 1C). Fourth, we selected among all the regions sampled in the Allen Human Brain Atlas Database the ROIs in which the selected genes would be analyzed (Figure 1D). We selected several neocortical regions in the temporal cortex because they have different function (Mesulam, 2023) and vulnerability to Alzheimer’s disease (Braak and Braak, 1996) and encompass different cortical types (García-Cabezas et al., 2020). Then, according to García-Cabezas et al. (2020), we assigned the corresponding cortical type value to each of the selected temporal cortical regions in the Allen Human Brain Atlas Database. We also selected the caudate nucleus region because it is a relatively homogeneous region of the brain [reviewed in Del Rey and García-Cabezas (2023)] that contrast with the variety of neuron types (Benavides-Piccione et al., 2021), layers (Sanides, 1975), and circuits (Vos de Wael et al., 2021) of the temporal cortex. Then, we mined the Allen Human Brain Atlas Database to collect the gene expression data of the selected genes in the selected brain ROIs. Finally, the collected data were conditioned prior to the statistical analysis (Figure 1E).

### 2.2. Cortical type analysis

We have explained the conceptual and practical aspects of cortical type analysis in our previous publications (García-Cabezas et al., 2020, 2022; Aparicio-Rodríguez and García-Cabezas, 2023). Briefly, cortical cells are arranged in layers that run parallel to the surface of the brain; these layers show systematic variation in their number and definition across gradients of progressive laminar complexification from the simple allocortex to the most complex koniocortex (García-Cabezas et al., 2020; John et al., 2022). Cortical types are defined along the laminar complexity gradient according to “the constant variations that one observes in each of the layers in different regions” of the cortex (von Economo and Koskinas, 1925/2008; von Economo, 1927/2009). The practical value of cortical types lies in their relation with synaptic plasticity (García-Cabezas et al., 2017), cortico-cortical connections (Barbas and Rempel-Clower, 1997; García-Cabezas et al., 2019; Aparicio-Rodríguez and García-Cabezas, 2023), cortical hierarchies (Mesulam, 1998; Chanes and Barrett, 2016; Hilgetag and Goulas, 2020), and the ontological and phylogenetic origin of the different parts of the cortex (García-Cabezas et al., 2022). We predicted that the expression of several genes related to glutamatergic and GABAergic transmission would vary gradually along the gradient of cortical types. These predictions are based on data obtained in postmortem macaque brains that suggest that the number of excitatory synapses in the primate cortical neuropil may decrease in parallel to laminar complexity, while the number of Parvalbumin inhibitory neurons increases (see Box 1). We also predicted that epigenetic regulation would vary gradually along the gradient of cortical types because areas of simple cortical type are likely endowed with higher synaptic plasticity and more flexible circuits to perform their functions related to learning and memory than areas of more complex types (see Box 1); we consider that flexible neuron function and higher synaptic plasticity should be related to flexible regulation of gene expression in the nucleus of neurons (and, probably, of glial cells).

For illustration, we show representative micrographs of cortical types and neurons taken from human temporal lobe sections stained with Nissl-technique. We also show representative micrographs of the human caudate nucleus for comparison with the cortex. The tissue from the human brain (neurotypical male, 58 year old) that was micrographed was used in previous studies (García-Cabezas et al., 2007) and its use for the present study was approved by the Ethics Committee for Research of Autonomous University of Madrid (Authorization CEI-104- 2011). Nissl technique was performed as described before (García-Cabezas et al., 2016) and micrographs were taken with a digital camera (DV-20, MicroBrightField, Europe) attached to a Zeiss Axioskop microscope (Oberkochen, Germany) using Neurolucida software (MicroBrightField, Colchester, VT, USA). Figures were assembled using PowerPoint software.

### 2.3. The Allen Brain Institute Human Brain Atlas database

The Allen Human Brain Atlas^1^ is a unique open-access multimodal atlas of the human brain that integrates anatomic and genomic information obtained using microarrays, *in situ* hybridization, and MRI. This atlas compiles data obtained from high-throughput analyses that used microarrays to quantify the expression of 1,000 genes (using more than 62,000 probes) in 500 samples per hemisphere (cerebrum, cerebellum, and brainstem) of six control individuals (Hawrylycz et al., 2012; Shen et al., 2012). Postmortem human tissue was processed for imaging, and subsequently, samples from approximately 900 anatomically defined sites were dissected for RNA isolation and microarray analysis. Five complete normal brains from male donors aged 24, 31, 39, 55, and 57 years and one from a female donor aged 49 were analyzed (Table 1). Samples from both hemispheres were pooled. Sample processing is described in detail in Hawrylycz et al. (2012).

**TABLE 1:** Allen Human Brain Atlas brain donor data.

Donor	Age	Sex	Ethnicity	Post-mortem interval (hours)
H0351.2001	24 years	M	Black or African American	23
H0351.2002	39 years	M	Black or African American	10
H0351.1009	57 years	M	White or Caucasian	26
H0351.1012	31 years	M	White or Caucasian	17
H0351.1015	49 years	F	Hispanic	30
H0351.1016	55 years	M	White or Caucasian	18

### 2.4. Selection of genes and regions of interest

In the present hypothesis driven study, we analyzed the expression of a series of genes related to glutamatergic transmission, GABAergic transmission, and epigenetic regulation in the human temporal cortex. First, we analyzed the expression of several genes related to glutamatergic and GABAergic transmission. The genes related to each neurotransmitter were classified into the following categories: synthetizing enzymes, degradation enzymes, ionotropic receptors, metabotropic receptors, and transporters. Then, the expression of several genes that codify enzymes involved in epigenetic modification of histones (acetylation, deacetylation, methylation, and demethylation) was analyzed. There are several epigenetic regulation mechanisms that promote or repress the readout of the DNA sequence. These mechanisms include histone posttranslational modifications, DNA methylation and acetylation, and RNA interference (Murr, 2010). To study epigenetic regulation in the human temporal cortex we analyzed the expression of histone-modifying enzymes; we chose this mechanism of epigenetic regulation because histone posttranslational modifications can be labeled on brain tissue using immunohistochemistry (García-Cabezas et al., 2018), allowing for correlation between enzyme gene expression and enzyme product in future studies.

The Gene Name, Gene Symbol, NCBI Accession Number, or Entrez Gene ID was searched for each selected gene in “NCBI gene.”^2^ The selected genes, their IDs, and their probes are tabulated in the Supplementary Table 1.

We analyzed the expression of the selected genes in several ROIs in the human temporal cortex and caudate nucleus. The temporal neocortex in the Allen Human Brain Atlas is parcellated into ten ROIs: temporal pole, parahippocampal gyrus, fusiform gyrus, inferior temporal gyrus, middle temporal gyrus, superior temporal gyrus, planum polare, planum temporale, transverse gyri, and Heschl’s gyrus (see text footnote 1). We selected six of these regions (temporal pole, parahippocampal gyrus, inferior temporal gyrus, middle temporal gyrus, superior temporal gyrus, and Heschl’s gyrus) that encompass all cortical types (García-Cabezas et al., 2020). We categorized the six selected cortical ROIs according to our previous cortical type analysis of the human cortex (García-Cabezas et al., 2020). ROIs were color coded: red for temporal pole, blue for parahippocampal gyrus, green for inferior temporal gyrus, purple for the middle temporal gyrus, orange for the superior temporal gyrus, black for the Heschl’s gyrus, and brown for the head of the caudate (Figure 1D).

The human temporal cortex has great diversity of neuron types (Benavides-Piccione et al., 2021), layers (Sanides, 1975), and circuits (Vos de Wael et al., 2021); more important, variations in cytology, structure, and connections across parts of the temporal cortex are not random, but change gradually in parallel to laminar complexification (García-Cabezas et al., 2020), as showed for cellular and molecular markers related to synaptic plasticity in the macaque frontal cortex (García-Cabezas et al., 2017, 2018). The systematic and gradual variation of temporal cortical areas contrasts with the relative homogeneity of neurons and circuits in the caudate nucleus; also, most neurons in the caudate are GABAergic, while inhibitory neurons are much less abundant in the cerebral cortex [reviewed in Del Rey and García-Cabezas (2023)]. For this reason, we used the *a priori* more homogeneous data from the caudate nucleus to contrast with the *a priori* more heterogenous data obtained from the six cortical temporal ROIs.

### 2.5. Data mining, collection, and conditioning

The Allen Human Brain Atlas allows for different search functions. In “Gene Search”, the gene of interest is entered and the result obtained comprises all the probes that hybridize to that gene and their expression in each of the brain regions of the donors. In “Differential Search”, gene expression in target regions and contrast regions is compared across donors of interest. Another interesting search option of the atlas is “Gene Classification” in which various genes have been categorized according to their relationship with pathologies or signaling pathways. Finally, there is the “Mouse Differential Search” option to compare data between human and murine brains.

In the present study we entered in “Gene search” the Gene Name, Gene Symbol, NCBI Accession Number, or Entrez Gene ID for the selected genes and obtained data for each of the probes hybridized to these genes in the ROIs. We collected the Z-Score value of each of the selected genes in the ROIs (in the temporal neocortex and caudate nucleus) and tabulated them using Excel software (Figure 1E). The Z-Score value is the number of standard deviations by which the value of an observed datum is higher or lower than the mean value of what is observed or measured (DeVore, 2017). If the Z-Score value is above the average, it has positive score and it will be showed in reddish tones in the maps of the Allen Human Brain Atlas; if it is below the average, it has negative score and will be showed in bluish tones in the maps of the Allen Human Brain Atlas.

For each selected gene, we obtained the Z-Score value for all the probes (see Excel file “ASV-AUH-MAGC-Data” in Supplementary Table 1) hybridized to the mRNA of that gene in the six study individuals and in each of the seven ROIs. The Z-Score values were collected and tabulated manually as showed in Figure 1E. First, the ROIs were entered in columns and the six brain donors were entered as subcolumns for each ROI. Second, the probe IDs of the selected genes were entered in rows. Finally, the Z-Score value for each probe obtained from each donor in each ROI was entered in the corresponding cell of the datasheet.

### 2.6. Statistical analysis and graph elaboration

The collected and conditioned data in the Excel spreadsheet were entered in GraphPad Prism software (Version 8.0.2, GraphPad Software) for statistical analysis. The data were entered as the mean of Z-Score value of all the probes for each gene in each ROI ± Standard Error of the Mean; if there was only one probe for one gene the Z-Score value of that probe was entered. We looked for differences in the Z-Score value of probes of the selected genes among the ROIs. A single-factor analysis was performed (one-factor ANOVA test with Tuckey’s multiple comparisons test) if only one probe was hybridized with a given mRNA. A two-factor test was performed, followed by multiple comparisons between the regions (two-factor ANOVA test with multiple comparisons), if two or more probes were hybridized with a given mRNA. The degrees of significance were set at *p* = 0.05: *p* < 0.05 (*), *p* < 0.01 (**), and *p* < 0.001 (***).

Graphs were elaborated with GraphPad Prism software. The data in the graphs corresponding to each ROI were color coded as in Figure 1D and represented in nested scatter plots with bars and plus points.

## 3. Results

### 3.1. Cortical types in the human temporal cortex

**FIGURE 2:**
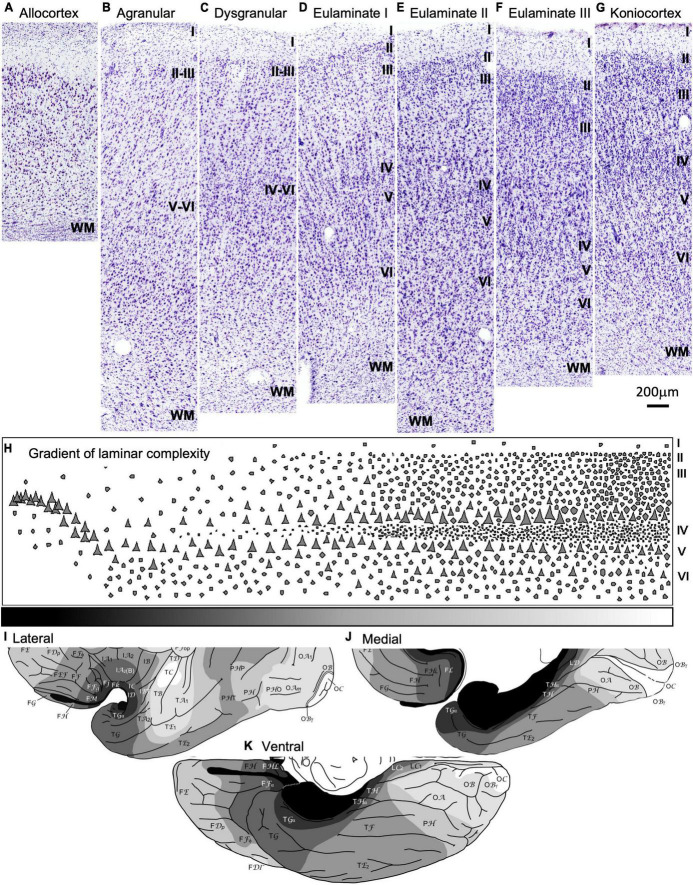
Laminar architecture of the human temporal cortex. **(A–G)** Micrographs show representative parts of the human temporal cortex for each cortical type from brain sections stained with Nissl. All cortical types (allocortical, mesocortical agranular, mesocortical dysgranular, eulaminate I, eulaminate II, eulaminate III, and koniocortex) are indicated on top of the corresponding micrograph. **(H)** Cartoon depicting the gradient of laminar complexifications from allocortical areas (left) to koniocortical areas (right). The gray scale (bottom) represents progressive laminar complexity (black: simplest laminar architecture; white: most complex laminar architecture). **(I–K)** Maps of the human tempral cortex (**I**, lateral view; **J**, medial view; **K**, ventral view) showing the distribution of cortical types in greyscale (black: simplest laminar architecture; white: most complex laminar architecture); these maps are modified from Figures 8A–C in García-Cabezas et al. (2020). Roman numerals indicate cortical layers. WM, white matter. Calibration bar below panel **(G)** applies to panels **(A–G)**.

The number of cortical layers and their definition is not constant throughout the human cerebral cortex. Laminar complexity increases from the simpler areas in the allocortex to the most complex koniocortical areas. Below, we briefly describe the progressive laminar complexification across the human temporal cortex as showed in the micrographs in Figures 2A–G.

In the human temporal cortex, the areas with the simplest laminar architecture are allocortical areas in the hippocampal formation and the primary olfactory cortex. These areas have 2–3 layers (Figure 2A) with a single layer of pyramidal projection neurons. In mesocortical areas adjacent to the hippocampus and primary olfactory cortex, the pyramidal projection neurons are arranged in superficial (II-III) and deep (V-VI) layers, the latter being more prominent; these areas lack granular layer IV and are called agranular (Figure 2B). Adjacent to agranular areas there are mesocortical dysgranular areas that have rudimentary and discontinuous layer IV (Figure 2C). Mesocortical areas are also called neocortical *non-sensu stricto* areas. The progression of temporal cortex laminar complexification proceeds with the appearance of a fully developed granular layer IV in isocortical areas, also called eulaminate or neocortical *sensu stricto* areas (Figures 2D–F). Eulaminate I areas have equally prominent superficial (II-III) and deep (V-VI) layers separated by a well-developed granular layer IV; these areas have pyramidal neuron bodies of comparable size in layers III and V (Figure 2D). In eulaminate II areas, the superficial (II-III) layers are more prominent than deep (V-VI) layers, granular layer IV is thicker than in eulaminate I areas, and the largest pyramidal neuron bodies are in layer III (Figure 2E). In eulaminate III areas, the largest pyramidal neuron bodies in layer III are larger and their superficial (II-III) layers are more prominent than in eulaminate II areas (Figure 2F). Finally, the areas with the most complex laminar architecture are koniocortical areas, which have the thickest layer IV and the most prominent superficial (II-III) layers.

Cortical types are categories in which regions with a comparable degree of laminar complexity are grouped together, regardless of their function, system, or location in the cerebral cortex (García-Cabezas et al., 2020). Because variation in laminar complexity is gradual (Figure 2H), there are no clear boundaries between cortical types along the cerebral cortex. Thus, cortical types constitute a continuum in the laminar gradient of complexification, which means that abrupt changes in laminar complexity will not be observed when moving from one adjacent cortical type to another. However, due to the need for categorization and comparison between studies, pragmatic boundaries between cortical types are defined. It is important to note that while two distant cortical areas may have a comparable or radically different cortical type, two adjacent cortical areas will always share a similar or close cortical type. Thus, areas that have a very different degree of laminar complexity cannot be adjacent (Figures 2I–K).

All cortical types (allocortical, mesocortical agranular, mesocortical dysgranular, eulaminate I, eulaminate II, eulaminate III, and koniocortex) are showed in greyscale (black: simplest laminar architecture; white: most complex laminar architecture) in maps of the human temporal cortex in Figures 2I–K [modified from Figures 8A–C in García-Cabezas et al. (2020)]. Accordingly, the cortical type of the six ROIs in the temporal cortex in the Allen Human Brain Atlas is described as follows: the parahippocampal gyrus and the temporal pole contain mesocortical (agranular and dysgranular) areas; the inferior temporal gyrus contains eulaminate I areas; the middle temporal gyrus contains eulaminate I, eulaminate II, and eulaminate III areas; the superior temporal gyrus also contains eulaminate areas but with a larger proportion of eulaminate III areas than the middle temporal gyrus; finally, the Heschl’s gyrus contains the primary auditory areas, which are koniocortical.

### 3.2. Predictions based on cortical type in the human temporal cortex

Areas of simple laminar architecture are at the top of cortical hierarchies, while eulaminate areas of progressively more complex laminar architecture are placed in progressively lower levels of cortical hierarchies with koniocortical areas at the bottom (Mesulam, 1998; Chanes and Barrett, 2016; Hilgetag and Goulas, 2020). In other words, areas of simper laminar architecture play “generalist” roles and areas of complex laminar architecture play “specialist” roles across cortical hierarchies (Barbas, 2015; Barbas et al., 2018). Thus, mesocortical areas should be endowed with higher synaptic plasticity to accomplish their function in memory and learning, as suggested by Barbas (1995). This hypothesis is supported by *postmortem* anatomical studies in macaques that suggest that synaptic plasticity may decrease in parallel to laminar complexity (see Box 1). Also, projection neurons in mesocortical areas, according to their “generalist” roles, should have larger receptive fields and, thus, larger dendritic arborization with more spines, to integrate the variety of afferents provided from areas in lower levels of cortical hierarchies. In contrast, as we move into progressively more complex laminar architecture, eulaminate projection neurons should have smaller receptive fields and, thus, smaller dendritic arborization with fewer spines. This hypothesis is supported by anatomical studies in macaques that show larger dendritic basolateral arborization in mesocortical compared to eulaminate pyramidal neurons [Elston et al., 2005; see also Table 2 in García-Cabezas et al. (2018)]. Studies in the human temporal cortex have also showed larger basolateral dendritic arbors with more spines in the pyramidal neurons of the mesocortical temporal pole than in eulaminate posterior temporal areas (Benavides-Piccione et al., 2021). Other anatomical studies of the human and non-human primate cortex point at structural differences in the neuropil of areas with simple vs. complex laminar architecture. For instance, several anatomical findings in macaques suggest that the number of excitatory synapses in the cortical neuropil may decrease in parallel to laminar complexity (see Box 1).

Taking into account all the observations summarized in the preceding paragraph, we predicted that the expression of genes related to glutamatergic transmission would be higher in areas of simpler laminar architecture (agranular and dysgranular cortical types) and would decrease in parallel to laminar complexification. This prediction is based on data suggesting decreasing numbers of excitatory synapses in parallel with laminar complexification (see Box 1). Also, we predicted that the expression of genes related to GABAergic transmission would be lower in areas of simpler laminar architecture and would increase in parallel to laminar complexification, because the number of inhibitory neurons (and, probably, inhibitory synapses) increases in parallel with laminar complexification (see Box 1). Finally, the integrative role of mesocortical areas due to their position at the top of cortical hierarchies (Mesulam, 1998; Chanes and Barrett, 2016; Hilgetag and Goulas, 2020) also suggest that their neurons should have higher synaptic plasticity (see Box 1) and also should be more plastic in their epigenetic regulation. Therefore, we predicted that the expression of genes related to posttranslational histone modification should be higher in areas of simpler cortical types and would decrease in parallel to laminar complexification.

### 3.3. Cytology of the human temporal cortex and caudate nucleus

The human cerebral cortex has a variety of neuron types whose neuron bodies and dendritic arborization also vary in size [reviewed in Peters and Jones (1984)]. First, there are projection neurons, which are excitatory and have spiny dendrites. Second, there are excitatory interneurons that have spiny dendrites; these neurons are more abundant in layer IV. Finally, there are inhibitory GABAergic interneurons that have smooth dendrites and can be sorted into three classes according to the expression of calcium binding proteins [parvalbumin, calbindin, and calretinin; DeFelipe (1997)].

**FIGURE 3:**
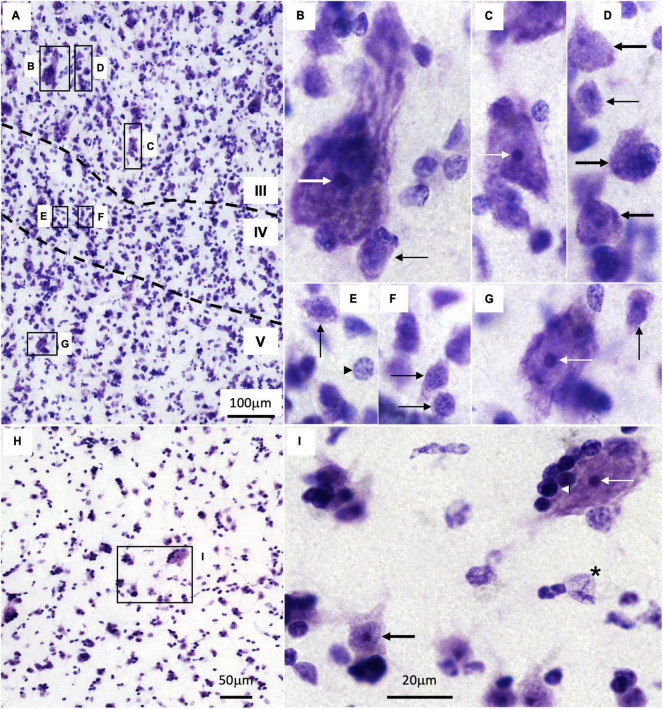
Cytology of the human temporal cortex and the caudate nucleus. **(A)** Low power micrograph of an eulaminate III area in the temporal cortex. Dotted lines mark the boundaries between cortical layers (indicated by Roman numerals); B**–**G indicate magnified insets in panels **(B–G)**. B–G High power micrographs of the corresponding insets in panel **(A)**. **(H)** Low power micrograph of the caudate; I indicates the magnified inset in panel **(I)**. I High power micrograph of the corresponding inset in panel **(H)**. Thick white arrow indicates the largest pyramidal neurons in layer III; thin white arrows indicate large body size neurons in layers III and V and in the caudate; thick black arrows indicate neurons with medium size bodies in layer III and medium spiny neurons in the caudate; thin black arrows indicate non-pyramidal neurons with small bodies in layers III, IV, and V; black arrowhead in panel **(E)** indicates an astrocyte; black and white arrowhead in panel **(I)** indicates an oligodendrocyte; black asterisk in panel **(I)** indicates an endothelial cell. Calibration bar in panel **(I)** applies to panels **(B–G,I)**.

Nissl staining does not reveal the dendrites and axons of neurons; it only labels the nuclei of neurons and glial cells and the body of neurons (García-Cabezas et al., 2016); thus, whether a cortical neuron is excitatory of inhibitory or is projection neuron or interneuron cannot be safely stated in Nissl stained sections. Still, Nissl technique reveals in the human cortex a large variety of neuron body sizes with specific nuclear architecture. The largest neuron bodies across cortical areas, except for Betz and Meynert cells in layer V of the primary motor and primary visual areas respectively (Lewis, 1878; Chan-Palay et al., 1974), are found in layer III of eulaminate III areas (Figure 3A; thick white arrow, Figure 3B; García-Cabezas et al., 2020). These largest pyramids are scattered among more abundant pyramids of large body size (thin white arrow, Figure 3C). Pyramidal neurons with medium size bodies are also found in layer III (thick black arrows, Figure 3D). In layer IV, there are abundant non-pyramidal small neurons bodies with scant rims of cytoplasm surrounding the neuronal nucleus (thin black arrows, Figures 3E, F). Such non-pyramidal small neurons are also found in less numbers in layers III and V (thin black arrows, Figures 3B, D, G). The largest neuron bodies in layer V (thin white arrow, Figure 3G) of eulaminate III areas can be as large as large pyramidal bodies in layer III (thin white arrow, Figure 3C). Areas of simpler cortical type have smaller neuron bodies in layer III and fewer non-pyramidal neurons in layer IV (García-Cabezas et al., 2018, 2020).

The size of the cortical neuron nucleus and nucleolus increases in parallel to neuron body size (Figures 3B–G; García-Cabezas et al., 2018). Also, heterochromatin grains are more abundant in the cell nucleus of neurons with small bodies (thin black arrows, Figures 3B, D–G) than with large bodies (thick and thin white arrows, Figures 3B, C, G).

In contrast to the cortex, the cytoarchitecture of the human caudate nucleus as visualized in Nissl-stained sections is quite homogeneous (Figure 3H) because GABAergic medium spiny neurons (MSNs) constitute the majority of striatal neurons (86% in macaques, Del Rey et al., 2022). In Nissl-stained sections, MSNs (thick black arrow, Figure 3I) appear as large as cortical medium size pyramids (thick black arrow, Figure 3D). Striatal interneurons, which can be cholinergic, GABAergic, or catecholaminergic, are less abundant (14% in macaques, Del Rey et al., 2022) than MSNs and are not distinguished in Nissl-stained sections, except for cholinergic interneurons whose neuron bodies are significantly larger than MSN bodies (Figures 3I, thin white arrow).

Intermixed with cortical and striatal neurons there are glial cells like astrocytes (black arrowhead, Figure 3E), oligodendrocytes (white and black arrowhead, Figure 3I), and microglia (not showed). There are also endothelial cells (black asterisk, Figure 3I). We have described previously the distinct cytological features in Nissl-stained sections of each of these non-neuron cell types (García-Cabezas et al., 2016).

The variety and density of cortical neurons and glial cell types changes quantitatively and qualitatively across layers and cortical types (Dombrowski et al., 2001; Zikopoulos et al., 2018). This variation must be taken into account for the analysis and interpretation of microarray gene expression data because this technique disintegrates brain tissue and mixes all cells together.

### 3.4. Gene expression data across temporal areas and the caudate nucleus

The results obtained in the analysis of the expression of genes related to glutamatergic and GABAergic neurotransmission as well as with posttranslational histone modification are detailed below.

#### 3.4.1. Expression of glutamatergic transmission related genes

**FIGURE 4:**
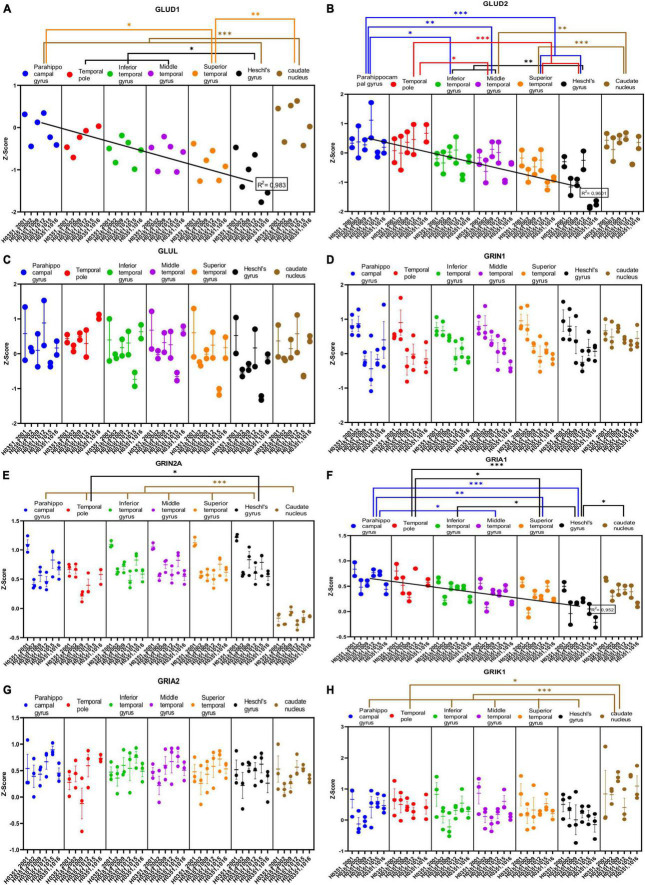
Expression of genes related to glutamatergic transmission in temporal cortex ROIs and the caudate nucleus. The Z-Score value of each gene is represented on the *y* axis. Each ROI is represented by a color: parahippocampal gyrus in blue, temporal pole in red, inferior temporal gyrus in green, middle temporal gyrus in purple, superior temporal gyrus in orange, Heschl’s gyrus in black, and caudate nucleus in brown. The six study individuals are represented on the *x* axis for each ROI. The solid circles represent the values for each of the probes that hybridize to the gene of interest for each individual and ROI. **(A)** Glutamate dehydrogenase 1 (GLUD1) synthetizing enzyme. **(B)** Glutamate dehydrogenase 2 (GLUD2) synthetizing enzyme. **(C)** Glutamate-ammonia ligase (GLUL) degradation enzyme. **(D)** Glutamate ionotropic receptor N-methyl-D-aspartic acid receptor type subunit 1 (GRIN1). **(E)** Glutamate ionotropic receptor N-methyl-D-aspartic acid receptor type subunit 2A (GRIN2A). **(F)** Glutamate ionotropic receptor α-Amino-3-hydroxy-5-methyl-4-isoxazolepropionic acid type subunit 1 (GRIA1). **(G)** Glutamate ionotropic receptor α-Amino-3-hydroxy-5-methyl-4-isoxazolepropionic acid type subunit 2 (GRIA2). **(H)** Glutamate ionotropic receptor kainate type subunit 1 (GRIK1). The degrees of significance were set at p = 0.05: *p* < 0.05 (*), *p* < 0.01 (**), and *p* < 0.001 (***).

**FIGURE 5:**
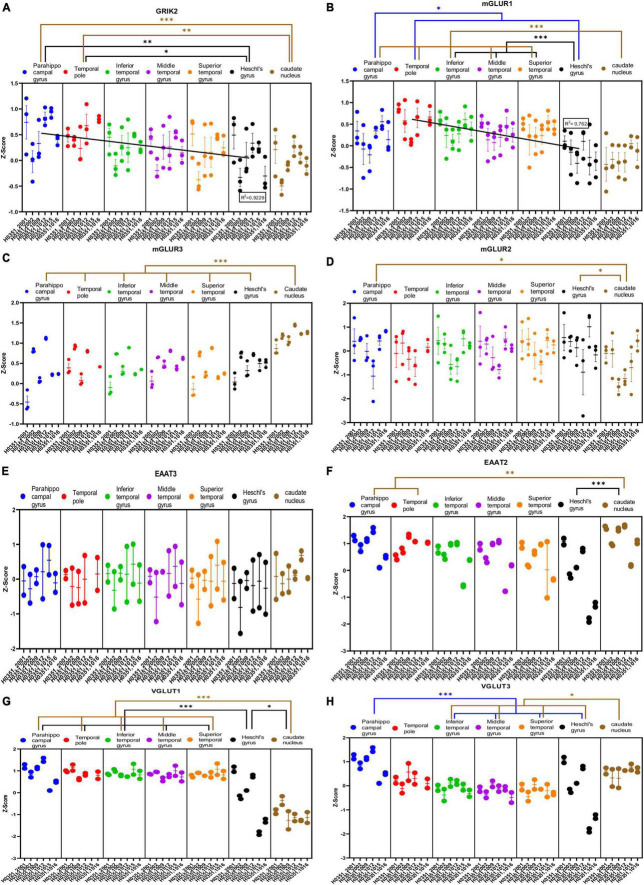
Expression of genes related to glutamatergic transmission in temporal cortex ROIs and the caudate nucleus. The Z-Score value of each gene is represented on the *y* axis. Each ROI is represented by a color: parahippocampal gyrus in blue, temporal pole in red, inferior temporal gyrus in green, middle temporal gyrus in purple, superior temporal gyrus in orange, Heschl’s gyrus in black, and caudate nucleus in brown. The six study individuals are represented on the *x* axis for each ROI. The solid circles represent the values for each of the probes that hybridize to the gene of interest for each individual and ROI. **(A)** Glutamate ionotropic receptor kainate type subunit 2 (GRIK2). **(B)** Glutamate metabotropic receptor 1 (mGluR1). **(C)** Glutamate metabotropic receptor 3 (mGluR3). **(D)** Glutamate metabotropic receptor 2 (mGluR2). **(E)** Excitatory amino acid transporter 3 (EAAT3). **(F)** Excitatory amino acid transporter 2 (EAAT2). **(G)** Vesicular glutamate transporter 1 (VGLUT1). **(H)** Vesicular glutamate transporter 3 (VGLUT3). The degrees of significance were set at *p* = 0.05: *p* < 0.05 (*), *p* < 0.01 (**), and *p* < 0.001 (***).

We analyzed the expression of several genes related to glutamatergic transmission in the caudate nucleus and six ROIs of the human temporal cortex (Figures 4A–H, 5A–H). Specifically, we analyzed the genes that encode two synthetizing enzymes [glutamate dehydrogenase 1 (GLUD1) and 2 (GLUD2)]; one degradation enzyme [glutamate-ammonia ligase (GLUL)]; six ionotropic receptor subunits [N-methyl-D-aspartic acid receptor subunits NR1 and NR2A (GRIN1 and GRIN2A), α-Amino-3-hydroxy-5-methyl-4-isoxazolepropionic acid receptor subunits (GRIA1 and GRIA2), and kainate receptor subunits (GRIK1 and GRIK2)]; three metabotropic receptors (mGluR1, mGluR2, and mGluR3); and the excitatory amino acid transporters 2 (EAAT2) and 3 (EAAT3), as well as the vesicular glutamate transporters 1 (VGLUT1) and 3 (VGLUT3).

Some of the analyzed genes (GLUD1, GLUD2, NR2A, GRIK1, mGluR1, mGluR3, VGLUT1, VGLUT3) were differentially expressed (*p* < 0.05) in the caudate nucleus compared to the temporal ROIs. The expression of these genes in the caudate nucleus was higher (GLUD1, GLUD2, GRIK1, mGluR3, and VGLUT3) or lower (NR2A, mGluR1, and VGLUT1) than in the cortical ROIs (Figures 4A, B, E, H, 5B, C, G, H).

Most genes did not show statistically significant differences in their expression across the cortical ROIs. However, the expression of GLUD1, GLUD2, GRIA1, GRIK2, and mGluR1 in temporal areas decreased (*p* < 0.05) in parallel to increased laminar complexity (Figures 4A, B, F, 5A, B).

#### 3.4.2. Expression of GABAergic transmission related genes

**FIGURE 6:**
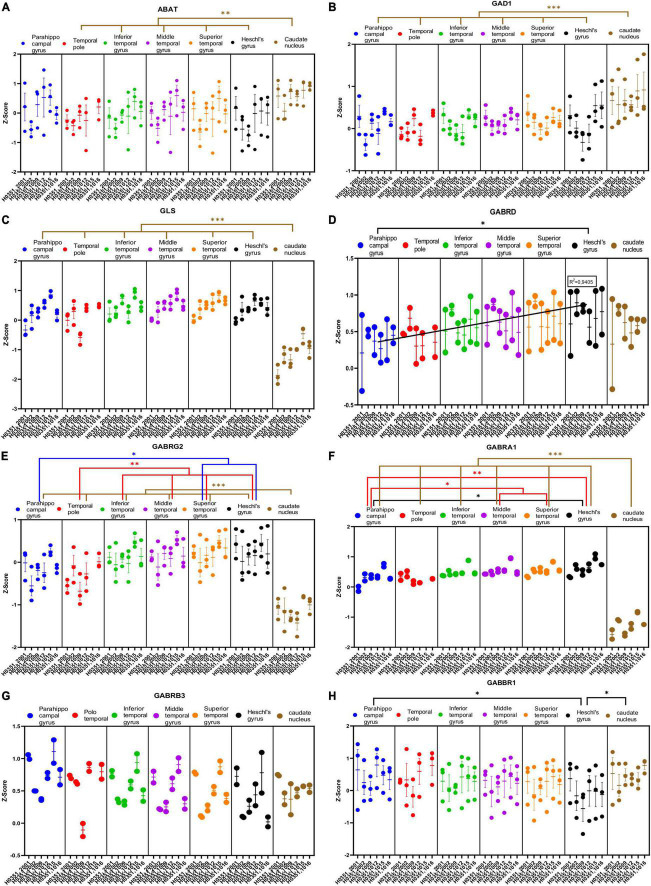
Expression of genes related to GABAergic transmission in temporal cortex ROIs and the caudate nucleus. The Z-Score value of each gene is represented on the *y* axis. Each ROI is represented by a color: parahippocampal gyrus in blue, temporal pole in red, inferior temporal gyrus in green, middle temporal gyrus in purple, superior temporal gyrus in orange, Heschl’s gyrus in black, and caudate nucleus in brown. The six study individuals are represented on the x axis for each ROI. The solid circles represent the values for each of the probes that hybridize to the gene of interest for each individual and ROI. **(A)** 4-aminobutyrate aminotransferase (ABAT). **(B)** Glutamate decarboxylase 1 (GAD1). **(C)** Glutaminase (GLS). **(D)** GABA ionotropic type A receptor subunit delta (GABRD). **(E)** GABA ionotropic type A receptor subunit gamma2 (GABRG2). **(F)** GABA ionotropic type A receptor subunit alpha1 (GABRA1). **(G)** GABA ionotropic type A receptor subunit beta3 (GABRB3). **(H)** GABA metabotropic type B receptor subunit 1 (GABBR1). The degrees of significance were set at *p* = 0.05: *p* < 0.05 (*), *p* < 0.01 (**), and *p* < 0.001 (***).

**FIGURE 7:**
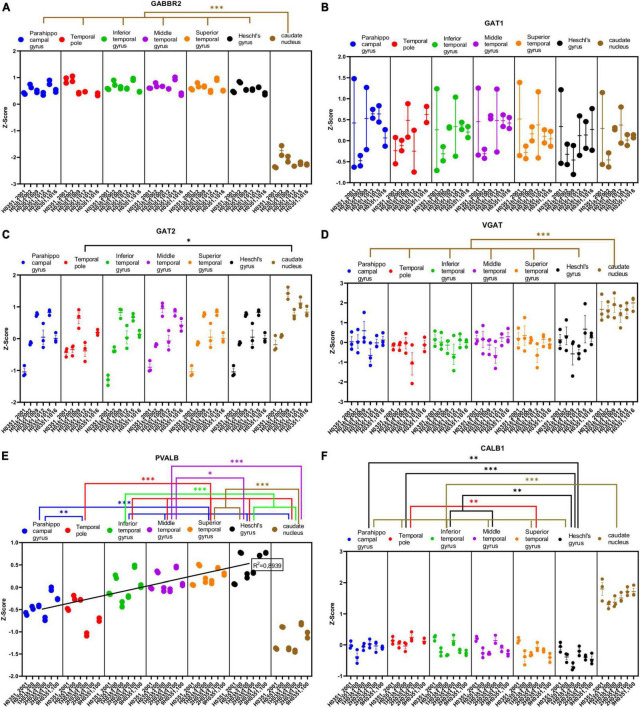
Expression of genes related to GABAergic transmission in temporal cortex ROIs and the caudate nucleus. The Z-Score value of each gene is represented on the *y* axis. Each ROI is represented by a color: parahippocampal gyrus in blue, temporal pole in red, inferior temporal gyrus in green, middle temporal gyrus in purple, superior temporal gyrus in orange, Heschl’s gyrus in black, and caudate nucleus in brown. The six study individuals are represented on the *x* axis for each ROI. The solid circles represent the values for each of the probes that hybridize to the gene of interest for each individual and ROI. **(A)** GABA metabotropic type B receptor subunit 2 (GABBR2). **(B)** GABA transporter 1 or GAT1 (SLC6A1). **(C)** GABA transporter 2 or GAT2 (SLC6A12). **(D)** Vesicular GABA transporter or VGAT (SLC32A1). **(E)** Parvalbumin (PALVB). **(F)** Calbindin (CALB1). The degrees of significance were set at *p* = 0.05: *p* < 0.05 (*), *p* < 0.01 (**), and *p* < 0.001 (***).

We analyzed the expression of several genes related to GABAergic transmission in the human temporal cortex and the caudate nucleus (Figures 6A–H, 7A–F). Specifically, we analyzed the genes that encode the degradation enzyme 4-aminobutyrate aminotransferase (ABAT); two synthetizing enzymes [Glutamate decarboxylase 1 (GAD1) and glutaminase (GLS)]; four ionotropic receptor subunits [delta (GABRD), gamma2 (GABRG2), alpha1 (GABRA1), and beta3 (GABRB3)]; two metabotropic receptor subunits [gamma-aminobutyric acid type B receptor subunit 1 (GABBR1) and 2 (GABBR2)]; and three transporters [GABA transporter type 1 (GAT1) and 2 (GAT2), and GABA vesicular transporter (VGAT)]. In addition, we analyzed the expression of the calcium binding proteins parvalbumin (PVALB) and calbindin (CALB1) genes, because they label subclasses of GABAergic neurons in the primate cerebral cortex (DeFelipe, 1997).

Half of the analyzed genes (ABAT, GAD1, GLS, GABRG2, GABRA1, GABBR2, VGAT, PVALB, and CALB1) where differentially expressed (*p* < 0.05) in the caudate nucleus compared to the temporal ROIs. The expression of some of these genes in the caudate was higher (ABAT, GAD1, VGAT, and CALB1) or lower (GLS, GABRG2, GABRA1, GABBR2, and PVALB) than in the cortical ROIs (Figures 6A–C, E, F, 7A, D–F).

As with glutamatergic related genes, most GABAergic related genes did not show statistically significant differences in their expression across the ROIs in the temporal cortex. However, the expression of GABRD and PVALB in temporal areas increased (*p* < 0.05) in parallel to laminar complexity (Figures 6D, 7E). An exception to this pattern was found for CALB1 whose expression significantly decreased (*p* < 0.05) in parallel to increased laminar complexity (Figure 7F); this could be due to the expression of calbindin in a significant population of glutamatergic projection neurons in the superficial layers of primate mesocortical areas (DeFelipe, 1997; Joyce et al., 2020).

#### 3.4.3. Expression of epigenetic regulation related genes

**FIGURE 8:**
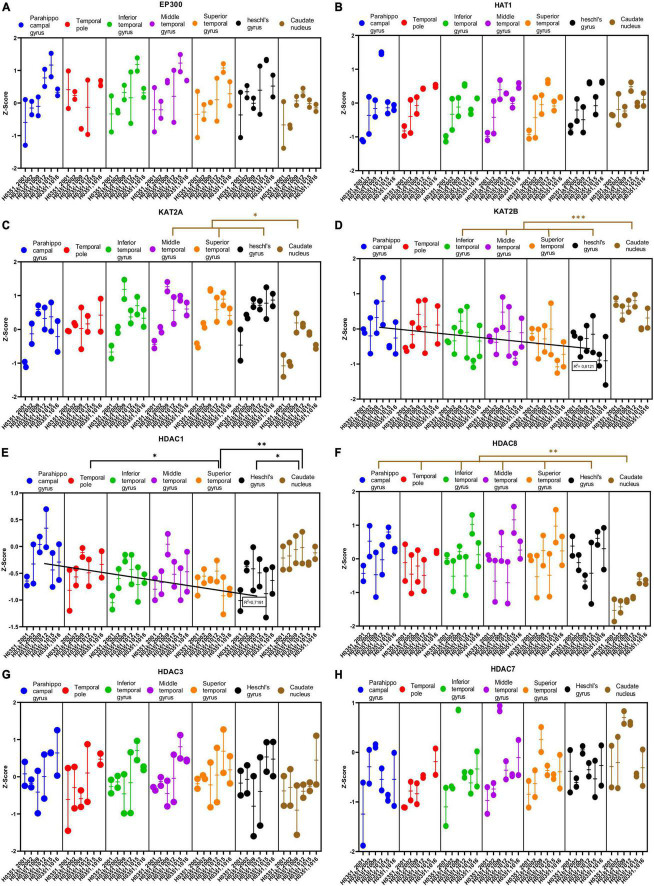
Expression of genes related to posttranslational histone modification in temporal cortex ROIs and the caudate nucleus. The Z-Score value of each gene is represented on the *y* axis. Each ROI is represented by a color: parahippocampal gyrus in blue, temporal pole in red, inferior temporal gyrus in green, middle temporal gyrus in purple, superior temporal gyrus in orange, Heschl’s gyrus in black, and caudate nucleus in brown. The six study individuals are represented on the *x* axis for each ROI. The solid circles represent the values for each of the probes that hybridize to the gene of interest for each individual and ROI. **(A)** Histone acetyltransferase EP300 (EP300). **(B)** Histone acetyltransferase 1 (HAT1). **(C)** Histone acetyltransferase KAT2A. **(D)** Histone acetyltransferase KAT2B. **(E)** Histone deacetylase 1 (HDAC1). **(F)** Histone deacetylase 8 (HDAC8). **(G)** Histone deacetylase 3 (HDAC3). **(H)** Histone deacetylase 7 (HDAC7). The degrees of significance were set at *p* = 0.05: *p* < 0.05 (*), *p* < 0.01 (**), and *p* < 0.001 (***).

**FIGURE 9:**
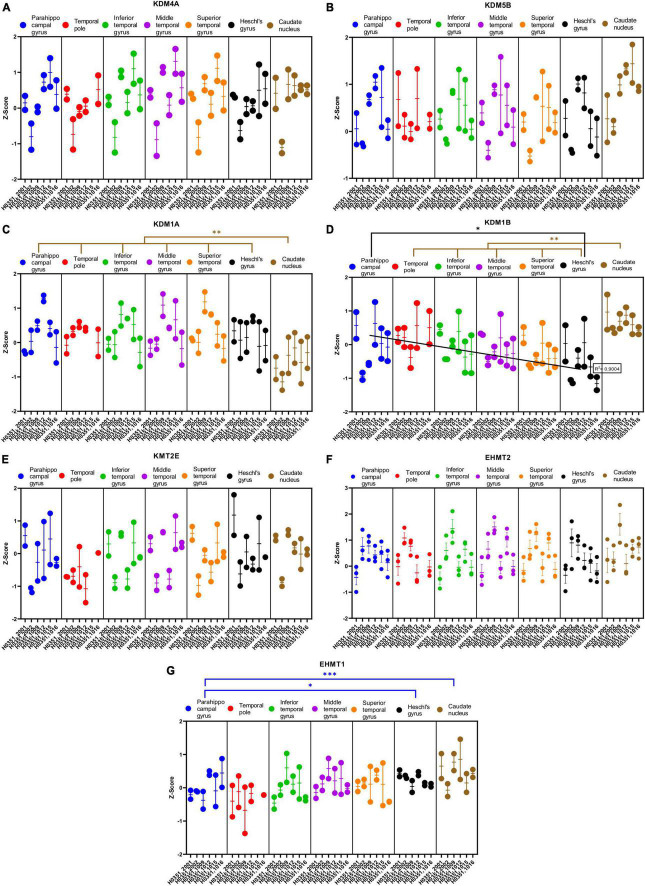
Expression of genes related to posttranslational histone modification in temporal cortex ROIs and the caudate nucleus. The Z-Score value of each gene is represented on the y axis. Each ROI is represented by a color: parahippocampal gyrus in blue, temporal pole in red, inferior temporal gyrus in green, middle temporal gyrus in purple, superior temporal gyrus in orange, Heschl’s gyrus in black, and caudate nucleus in brown. The six study individuals are represented on the x axis for each ROI. The solid circles represent the values for each of the probes that hybridize to the gene of interest for each individual and ROI. **(A)** Lysine demethylase 4A (KDM4A). **(B)** Lysine demethylase 5B (KDM5B). **(C)** Lysine demethylase 1A (KDM1A). **(D)** Lysine demethylase 1B (KDM1B). **(E)** Lysine methyltransferase 2E (KMT2E). **(F)** Euchromatic histone lysine methyltransferase 2 (EHMT2). **(G)** Euchromatic histone lysine methyltransferase 1 (EHMT1). The degrees of significance were set at *p* = 0.05: *p* < 0.05 (*), *p* < 0.01 (**), and *p* < 0.001 (***).

We analyzed the expression of several genes related to epigenetic regulation in the temporal cortex and the caudate nucleus (Figures 8A–H, 9A–G). Specifically, we analyzed four genes that encode histone acetylation enzymes [Histone acetyltransferase EP300 (EP300), Histone acetyltransferase 1 (HAT1), Histone acetyltransferase KAT2A (KAT2A), and Histone acetyltransferase KAT2B (KAT2B)]; four related to histone deacetylation [Histone deacetylase 1 (HDAC1), Histone deacetylase 3 (HDAC3), Histone deacetylase 7 (HDAC7), and Histone deacetylase 8 (HDAC8)]; four genes that encode histone demethylation enzymes [Lysine demethylase 4A (KDM4A), Lysine demethylase 5B (KDM5B), Lysine demethylase 1A (KDM1A), and Lysine demethylase 1B (KDM1B)]; and finally, three genes that encode histone methylation enzymes [Lysine methyltransferase 2E (KMT2E), Euchromatic histone lysine methyltransferase 1 (EHMT1), and Euchromatic histone lysine methyltransferase 2 (EHMT2)].

Some of the analyzed genes (KAT2B, HDAC1, HDAC8, KDM1A, and KDM1B) where differentially expressed (*p* < 0.05) in the caudate nucleus compared to the temporal ROIs. The expression of some of these genes in the caudate was higher (KAT2B, HDAC1, and KDM1B) or lower (HDAC8 and KDM1A) than in the cortical ROIs (Figures 8D–F, 9C, D).

Like with glutamatergic and GABAergic markers, most epigenetic markers genes did not show statistically significant differences in their expression across the ROIs in the temporal cortex. However, the expression of KAT2B, HDAC1, and KDM1B in temporal areas decreased (*p* < 0.05) in parallel to increased laminar complexity (Figures 8D, E, 9D).

## 4. Discussion

**FIGURE 10:**
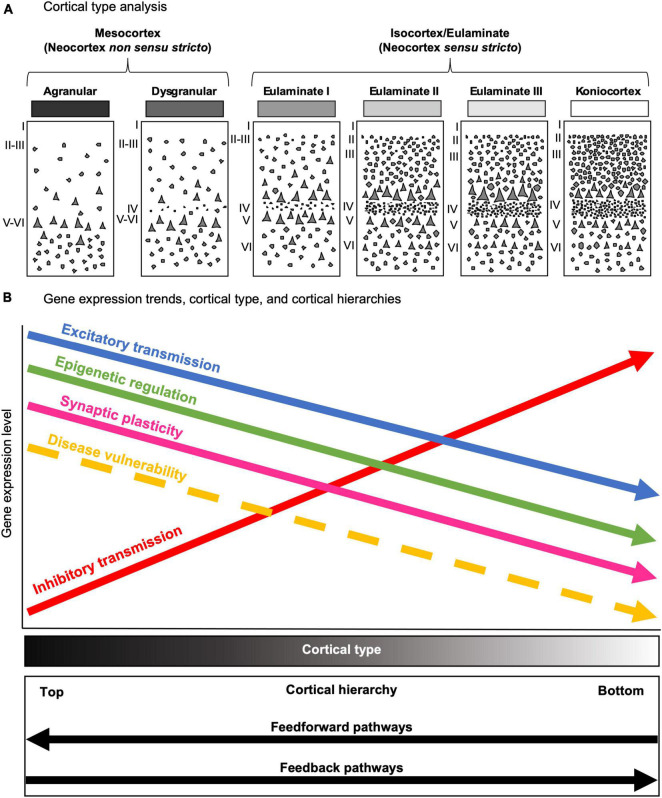
The predictive value of cortical type in the human cerebral cortex. **(A)** Cartoons depicting the progressive complexification of laminar architecture from mesocortical agranular (left) to koniocortical (right) types. All cortical types (mesocortical agranular, mesocortical dysgranular, eulaminate I, eulaminate II, eulaminate III, and koniocortex) are represented in greyscale (black: simplest laminar architecture; white: most complex laminar architecture). **(B)** Gene expression trends in parallel to cortical type, and cortical hierarchies. The *x* asis represents the levels of gene expression; the disease vulnerability trend is also represented with a dasehd arrow. The *y* axis represents laminar complexity, as showed by the Cortical type gray scale bar (black: simplest laminar architecture; white: most complex laminar architecture); the bottom box represents cortical hierarchy processing in parallel with laminar complexity according to Mesulam (1998), Barbas (2015), Chanes and Barrett (2016) and García-Cabezas et al. (2019). Excitatory transmission, epigenetic regulation turnover, synaptic plasticity, and selective vulnerability to diseases decrease from areas of simple laminar architecture to progressively more eulaminate types. In contrast, inhibitory transmission increases in parallel to laminar complexity.

In the present study we aimed to exemplify the importance of selecting the proper biological context in the human brain for gene expression data analysis and interpretation. Thus, we used cortical type as conceptual tool to make predictions about gene expression in the human temporal cortex and tested them with data from the Allen Human Brain Atlas. Our hypotheses were that the expression of several genes related to glutamatergic transmission, GABAergic transmission, and epigenetic regulation would vary gradually along the gradient of cortical types. Most of the genes analyzed did not show significant expression differences between temporal cortical ROIs, but many of them showed significant increases or decreases with respect to the caudate nucleus. Interestingly, the expression of a significant set of genes did vary across cortical temporal ROIs in agreement with the predicted gradual expression along the gradient of laminar complexity of the human cortex (Figure 10). Our results show that cortical type helps guiding the analysis and biological interpretation of high-throughput gene expression data obtained in the human cerebral cortex. Below, we discuss several aspects of gene expression in the human temporal cortex in relation to cortical function and selective vulnerability to neurological diseases.

### 4.1. Gene expression data support the flexible role of human mesocortical areas

We observed decreased expression of some genes related to glutamatergic transmission (GLUD1, GLUD2, GRIA1, GRIK2, and mGluR1) in temporal cortical ROIs in parallel to increased laminar complexity. In contrast, for two GABAergic genes (GABRD and PVALB) we found a trend of increased expression in temporal cortical ROIs in parallel with increased laminar complexity. In the case of CALB1, a calcium binding protein that labels a subset of cortical inhibitory interneurons in primates (DeFelipe, 1997), we observed a decrease of its expression in parallel to increased laminar complexity; this could be due to the fact that CALB1 also labels a significant population of excitatory pyramidal projection neurons in primate mesocortical areas (DeFelipe, 1997; Joyce et al., 2020).

The higher expression of glutamatergic receptors in areas of simpler laminar architecture could be due to higher numbers of excitatory synapses in the neuropil of these areas (blue arrow, Figure 10B) compared to areas of more complex laminar architecture; this hypothesis is supported by quantitative studies that show progressive increase in neuron density and intracortical myelin in primates in parallel with cortical complexification (Dombrowski et al., 2001; Zikopoulos et al., 2018; John et al., 2022), suggesting that the neuropil in mesocortical areas has more room for synapses compared to eulaminate areas (see Box 1). The majority of these synapses could be excitatory because they are the dominant type of synapse in the cerebral cortex (Peters et al., 1991). Also, pyramidal neurons in mesocortical human temporal areas have larger dendritic basolateral arborization and more spines than pyramidal neurons in eulaminate areas (Benavides-Piccione et al., 2021); more dendritic spines in mesocortical areas also support the hypothesis of more excitatory synapses in the mesocortical neuropil because spines are the typical postsynaptic element of excitatory synapses in the cerebral cortex (Peters et al., 1991).

The higher expression of the GABAergic genes GABRD and PVALB in areas of more complex laminar architecture could be due to higher numbers of inhibitory synapses and inhibitory neurons compared to areas of simpler laminar architecture (red arrow, Figure 10B), which are compatible with unbiased quantifications of PVALB expressing interneurons in the primate cortex (Dombrowski et al., 2001; García-Cabezas et al., 2017; Joyce et al., 2020). Altogether, the gene expression data presented in the present article suggest that simpler cortical types may have greater glutamatergic excitability compared to more complex types; on the other hand, complex cortical types seem to have greater GABAergic inhibitory control compared to simpler types.

Finally, the expression of some genes related to epigenetic modifications (KAT2B, HDAC1, and KDM1B) was higher in mesocortical areas and decreased as laminar complexity increased. This could suggest that histone modification turnover is higher in the cellular nucleus of neurons (and, probably, glial cells) in mesocortical areas than in eulaminate areas (green arrow, Figure 10B).

The results summarized in the preceding paragraphs point at relevant anatomical properties of cortical types that may be directly implied in the functional differentiation of the cortical networks arranged along cortical hierarchies. For instance, higher glutamatergic excitability is consistent with the “generalist” role of mesocortical areas. These areas are at the top of cortical hierarchies, integrate afferences form unimodal and multimodal areas of all sensory modalities, and are key in memory and learning (Barbas, 1995, 2015; Mesulam, 1998; Chanes and Barrett, 2016). Also, mesocortical pyramidal neurons have larger basolateral dendritic arborizations (Elston et al., 2005), consistent with larger receptive fields needed for higher integrative capacity. Thus, in order to adapt to changes, cortical areas of simple laminar architecture must be more excitable and more plastic and flexible, both at the synapse (pink arrow, Figure 10B) and the epigenetic regulation level (green arrow, Figure 10B), than eulaminate areas. In contrast, eulaminate areas of progressively more complex laminar architecture play a more “specialist” role, their neurons have smaller basolateral dendritic arborizations (Elston et al., 2005), and are less excitable with higher inhibition needed for lateral inhibition. Eulaminate areas must also be more stable, with lower synaptic plasticity, and less flexible epigenetic regulation.

Anyway, these hypotheses are supported but cannot be confirmed by our data. It must be considered that gene expression data does not relate linearly and strictly with cellular physiology. For instance, other epigenetic mechanisms, like RNA interference, could be functioning at a higher level in eulaminate than in mesocortical areas. Also, higher gene expression of histone-modifying enzymes or glutamate receptors does not equal higher levels of the real effectors of these genes, which are the enzyme and receptor proteins. Thus, the data presented in the present paper support the notion that glutamatergic transmission, GABAergic transmission, and epigenetic regulation vary in parallel to laminar complexity; buy only *in vivo* functional studies in human and non-human primates could confirm or discard these hypotheses.

### 4.2. Gene expression data in the human temporal cortex and selective vulnerability

Mesocortical areas in the temporal lobe show higher vulnerability to several neurological diseases, like Alzheimer’s disease (Braak and Braak, 1991), epilepsy (Frazzini et al., 2022), and viral encephalitis (Damasio and Van Hoesen, 1985), compared to temporal eulaminate areas (yellow dashed arrow, Figure 10B). What are the cellular and molecular factors that underlie these differences in vulnerability across cortical areas? Previously, we hypothesized that the potentially higher synaptic plasticity of mesocortical areas could be related to their higher vulnerability (García-Cabezas et al., 2017). The excitatory trend supported by data obtained in the present article (blue arrow, Figure 10B) could also be related to selective vulnerability of mesocortical areas because chronic glutamate excitotoxicity is a pathogenetic mechanism involved in several neurological diseases (Lewerenz and Maher, 2015). Accordingly, an excess of excitatory synapses in areas of simpler cortical architecture, as suggested in the present study, may increase extracellular concentration of glutamate and could underlie the selective vulnerability of mesocortical areas in the temporal lobe to neurodegeneration and other diseases. Lack of inhibition is also related to epilepsy (Khazipov, 2016); thus, the decreasing inhibitory trend (red arrow, Figure 10B) from areas of simple laminar architecture to progressively more eulaminate areas may also underlie the selective vulnerability of medial temporal areas to epilepsy.

The decreasing trend of epigenetic regulation in parallel to increasing laminar complexity (green arrow, Figure 10B), described in the present article for the first time, suggest that there are epigenetic factors acting in the cellular nucleus of neurons (and, probably, glial cells) in mesocortical areas that may underlie selective vulnerability to neurological diseases. This interesting hypothesis could be addressed in future studies sampling the human cerebral cortex of neurotypical and diseased brains with more powerful transcriptomic and epigenomic techniques; as showed in the present article, such future studies would be more successful in identifying biologically significant differences if they are designed using cortical type as guiding conceptual tool.

### 4.3. The opposite relationship between laminar and functional complexity

In the present article and in previous publications we use the term “laminar complexity” in reference to different degrees of cerebral cortex structural organization as examined in Nissl-stained sections under optical microcopy (Figures 2A–G). A look at the micrographs in Figure 2B (mesocortical agranular) and 2G (koniocortical) shows several structural features that define the “simpler laminar architecture” of mesocortical areas compared to the “more complex laminar architecture” of koniocortical areas. These structural features include fewer layers in mesocortical agranular (they lack layer IV) than in koniocortical areas; poor differentiation between layers II and III (and V and VI) in mesocortical agranular compared to koniocortical areas; and irregular boundaries between layers (*e. g.*, between layers I and II) in mesocortical areas compared to the more regular boundaries in koniocortical areas. In the vertical dimension, neurons are more dispersed in mesocortical areas, while in koniocortical areas they are arranged in observable vertical columns. Such “cytoarchitectonic columns” are in part due to the separation of neurons by bundles of myelinated axons that are more abundant and much thicker in koniocortical than in mesocortical agranular areas (García-Cabezas et al., 2019, 2020). As noted by Helen Barbas and her associates, the structural definition of columns across cortical areas is not uniform, but varies gradually in parallel to laminar complexity (Barbas et al., 2022). In summary, “laminar complexity” is defined and measured according to cytoarchitectonics.

The gene expression data in the present article support the idea that laminar complexity has an inverse relationship with markers that favor synaptic plasticity (García-Cabezas et al., 2017), with the length and number of spines of pyramidal neuron basolateral dendrites [Elston et al., 2005; see also Table 2 in García-Cabezas et al. (2018)], and with the expression of histone modification enzymes (Figures 8, 9). Altogether, laminar complexity in the primate cerebral cortex seems to be anticorrelated with synaptic plasticity, the number of excitatory synapses in the neuropil, pyramidal neuron receptive field size, and epigenetic turnover. Thus, areas with simpler laminar architecture seem to have more complex synaptic circuitry than eulaminate areas, along a gradient of circuit complexity anticorrelated with laminar complexity. Such circuit complexity gradient would support the progressive complexification of sensory representations from koniocortical to mesocortical areas. For instance, neurons in primary (koniocortical) sensory areas are responsive to simple stimuli, like angled bars; neurons in unimodal associative (eulaminate III and II) areas are responsive to more complex stimuli of just one modality; neurons in multimodal associative (eulaminate II and I) are responsive to more than one sensory modality; and, finally, neurons in paralimbic (mesocortical) areas are responsive to the emotional content and value of the stimuli (Mesulam, 1998; Barbas, 2015; Chanes and Barrett, 2016; García-Cabezas et al., 2019).

In summary, several gradients are identified along the primate (and, probably, along the mammalian) cerebral cortex: a laminar complexity gradient, a hierarchical processing gradient, a synaptic plasticity gradient, an excitatory gradient, an inhibitory gradient, and an epigenetic turnover gradient (Figure 10). The correlation and anticorrelation of these gradients provide an integrative point of view of cortical function and structure that can guide further research in normal and pathological human cerebral cortex studies; also, these gradients pose the question of how the primate cerebral cortex has been tangentially expanded to generate all these parallel levels of complexity, a question than is only partially answered (Puelles et al., 2019; García-Cabezas et al., 2022).

### 4.4. Conclusion: cortical type is a multifunction conceptual tool

Cortical type has been proved to be a useful conceptual tool for predicting the laminar patterns of connections between cortical areas in non-human primates (Barbas and Rempel-Clower, 1997; García-Cabezas et al., 2019; Aparicio-Rodríguez and García-Cabezas, 2023), which also predicts the position of areas along cortical hierarchies (Chanes and Barrett, 2016; Hilgetag and Goulas, 2020). Even more, cortical type allows for extrapolating data from non-human primates to humans with sound homology hypotheses (García-Cabezas et al., 2022). Cortical type is also related to the degree of synaptic plasticity (García-Cabezas et al., 2017) and, as suggested in the present article, also seems to be related with the epigenetic turnover in the cellular nucleus of cortical cells (green arrow, Figure 10B) and with the intensity of glutamatergic transmission (blue arrow, Figure 10B). Altogether, cortical type is also a good predictor of the selective vulnerability (yellow dashed arrow, Figure 10B) to several types of diseases that involve the cerebral cortex.

In summary, studies of the human cerebral cortex (from *in vivo* connectivity imaging to postmortem transcriptomics) could benefit from the use of cortical type as a powerful conceptual tool for experimental design. For instance, the selection of cortical samples from postmortem human brains for high-throughput gene expression techniques should be based on cortical type analysis. Researchers must keep in mind that a careful experimental design in a meaningful biological context will affect the downstream analysis of the data and their ability to identify biologically significant differences and discoveries.

## Data availability statement

The original contributions presented in this study are included in this article/Supplementary material, further inquiries can be directed to the corresponding author.

## Author contributions

MÁG-C developed the study conception and design and secured the funding. AU-H cut, stained, and photographed the human brain tissue used in Figures 2, 3. AU-H and MÁG-C analyzed the human brain Nissl-stained sections. AS-V mined the Allen Human Brain Atlas database and collected the gene expression data into excel spreadsheet. MÁG-C and AS-V analyzed the gene expression data. All authors assembled the figures, contributed to writing the manuscript, and approved its final version.

## Footnotes

1
https://human.brain-map.org/

2
https://www.ncbi.nlm.nih.gov/gene
